# Passive Control in a Continuous Beam under a Traveling Heavy Mass: Dynamic Response and Experimental Verification

**DOI:** 10.3390/s24020573

**Published:** 2024-01-16

**Authors:** George D. Manolis, Georgios I. Dadoulis

**Affiliations:** Laboratory for Experimental Strength of Materials and Structures, School of Civil Engineering, Aristotle University of Thessaloniki, GR-54124 Thessaloniki, Greece; dadoulis@civil.auth.gr

**Keywords:** bridges, travelling mass, vibrations, passive control, tuned mass damper, secondary systems, experimental verification

## Abstract

The motion of a heavy mass on a bridge span causes vibrations whose magnitude and frequency content depend on the mechanical properties of the structural system, including the magnitude of that mass and its speed of traverse. In order to limit vibrations that could potentially cause damage, a simple passive device configuration, namely the tuned mass damper (TMD), is introduced and its effect on the beam vibrations analyzed. Specifically, a TMD in the form of a single-degree-of-freedom (SDOF) unit comprising a mass and a spring is placed on the span to act as a secondary system for absorbing vibrations from the primary system, i.e., the bridge itself. A Lagrangian energy balance formulation is used to derive the governing equations of motion, followed by an analytical solution using the Laplace transform to investigate the transmission of vibratory energy between primary and secondary systems. Results are given in terms of time histories, Fourier spectra and spectrograms, where the influence of the TMD in reducing vibratory energy is demonstrated. The TMD is placed in the region where the beam’s transverse motion is at a maximum, while its mechanical properties are sub-optimal, in the sense that there is no separate damper present and minimal damping is provided by the spring element itself. In parallel with the analysis, a series of experiments involving a simply supported model steel bridge span traversed by a heavy mass are conducted to first gauge the analytical solution and then to confirm the validity of the proposed passive scheme.

## 1. Introduction

Tuned mass damper (TMD) technology has its origins in the early 1900s [1] and is characterized by mechanical simplicity, cost effectiveness and reliability. The two basic fields of application are civil engineering for motion control (bridges, towers, tall buildings) and mechanical engineering for vibration suppression in turbines and various types of machines. Early studies in optimized TMD design for harmonic motions can be traced to [2] and random excitations to [3]. In principle, TMDs can completely absorb vibrations at the selected tuned frequency, while material damping in the primary structure to which they are attached also plays a significant role in this respect, as it increases the frequency bandwidth of the response in the vicinity of the tuned frequency [4].

Standard TMD design [5] is basically an SDOF dynamic system comprising a spring and a damper connected to a small mass. As such, it is viewed as a secondary system attached to a primary system, i.e., to the structure itself. In terms of analysis, the latter may be represented as either a multi-degree-of-freedom (MDOF) system or as a continuous system. In either case, TMDs operate very close to the dominant mode of vibration of the primary structure, resulting in a substantial reduction in its dynamic responses. However, this vibration minimization is also dependent on the frequency content of the external loads, which implies that optimization is required in order for the TMD to operate in the most sensitive frequency band. Alternatively, nonlinear TMDs may be designed [6] which exhibit a variable (i.e., load dependent) natural frequency. A classification [7] of basic TMD technology distinguishes between (i) composite TMD design, (ii) distributed TMD design, (iii) MDOF TMD design and (iv) impact dampers in conjunction with a TMD. In terms of more recent applications, we mention a numerical investigation of the performance of a TMD in controlling the torsional vortex phenomenon for an aerodynamically streamlined twin-box girder suspension bridge [8]. The TMD parameters were gauged in terms of controlling the torsional response of the suspension bridge, and an effective range was reached and compared with the output provided by a genetic algorithm. Among recent work on the use of TMDs for vibration suppression, we mention [9], which examined a suspension bridge under the combined effect of wind and traffic [10], which used the theory of generalized functions to formulate and solve the equations of motion for a viscoelastic Bernoulli–Euler beam with rotational joints and multiple supports under a moving force [11], on the installation of two TMDs in a footbridge for pedestrian use, and [12], which examined the coupled bridge-moving vehicle system with the possibility of using the latter component as a TMD.

As previously mentioned, for a discrete primary structure representation, the design philosophy is based on the dominant mode as derived from a finite element analysis. When considering a continuous mass distribution (i.e., a waveguide) model for the primary structure with an attached TMD, a number of modeling issues arise. In this case, the governing differential equations of motion are obtained using either the Lagrangian or extended Hamilton’s principle [13]. It is then possible to proceed with an eigenvalue analysis to separate the modes of the combined system and tune the TMD accordingly. As far as TMD placement is concerned, most research articles focus on either cantilever beams or on beams with symmetric boundaries and geometric conditions. Thus, TMDs are placed at the top of the former category of structures and in the central span of the latter category, since this is where the dominant first vibration mode will yield maximum values for the kinematic variables. Although the basic design concept behind a TMD system is quite simple, its damping and stiffness parameter values are often determined through optimization [14] by defining a suitable objective function with appropriate design constraints. For random loads, the variance in response can be selected as the objective function, while for stationary random loads, the power spectral density (PSD) function of the structural response can be used [15]. Among the various solution strategies, we mention gradient-based optimization and global optimization.

In work published over the last thirty years, the focus has been on design objectives, on the effect of the mass ratio between primary and secondary structures, on different types of TMD designs and on the introduction of semi-active control mechanisms [7]. Recent developments in TMD technology have focused on refining TMD design beyond the simple SDOF system such as (i) the attachment of a secondary beam structure; (ii) ball damper or pendulum types of dampers; (iii) semi-active/active TMD systems; (iv) tuned liquid column dampers; (v) variable orifice hydraulic actuators; (vi) active variable stiffness dampers; (vii) electrorheological/magnetorheological fluid dampers; and (ix) magnetorheological elastomer–tuned vibration absorbers. Also, recently, a 2DOF nonlinear energy sink was proposed to suppress the vibration of a simply supported beam subjected to large-amplitude excitation in the vicinity of its fundamental frequency [6]. More specifically, the governing equation of the combined Bernoulli–Euler beam plus the nonlinear energy sink system was treated by Galerkin’s method, with the beam displacement written in terms of the generalized coordinates and the eigenfunctions of the stand-alone beam. The dimensionless equation of motion, after applying normality conditions along with the Euler–Lagrange equation, was solved by the complexification-averaging method, which is an approximate analytical solution for a two eigenmode expansion. This solution was compared with a numerical solution of the governing ordinary differential equations of motion using a Runge–Kutta method solver. In terms of results, it was found that at low excitation amplitudes, the SDOF nonlinear energy sink was more effective than 2DOF configuration in terms of resonant peak suppression and energy dissipation, while the reverse held true as the excitation amplitude increased. Finally, at high values of the nonlinear energy sink parameters, excitations make the system chaotic, but added damping reduces the unstable band and provides a stable response. Recent work along these lines is presented in [16], suggesting the possibility of placing electromagnetic energy harvesters in a multi-story building under random vibrations as a means for converting vibratory energy into electric current and in [17], proposing energy-regenerative TMDs placed in structures for power harvesting that could be used for running sensors for structural health monitoring purposes.

Regarding the vibration control of structures under the influence of moving loads, [18] recently investigated the tuned mass inerter system (TMIS) as an alternative lightweight passive control device, which contains a suspended mass, a parallel-connected tuned spring and an inerter-based subsystem. The study focused on optimizing the TMIS for the vibration suppression of multi-span beams under moving load series using the Bubnov–Galerkin integration method. This was carried out in conjunction with modal superposition using a few pre-specified modes of the combined beam-TMIS system under a moving load series. Thus, a design strategy was proposed to achieve a targeted performance by decreasing the moving-load-induced resonant response. The optimization algorithm used the tuned mass ratio as the objective function, plus the dynamic response amplitude under different speed parameters as constraint conditions for a coupled single-span, simply supported beam with the attached TMIS. In sum, the design TMIS was tuned to the dominant mode of the primary structure in order to be effective.

The purpose of the present work is to investigate the dynamic response of a combined structural system comprising an SDOF oscillator placed near the center of a simply supported beam to act as a TMD for controlling vibrations, due to the passage of a heavy point mass. The key considerations in this SDOF design are (i) a mass that is at least an order of magnitude less than that of the beam and (ii) a natural frequency that is tuned close to the dominant frequency of the supporting system. Despite the fact TMDs have been in use for many decades now in buildings, bridges, pylons, etc., for minimizing the response of these structures to dynamic loads such as ground motions and wind-induced pressure, the control of a continuous dynamic system subjected to a heavy moving mass has not been thoroughly examined. The reason is that a heavy moving mass modifies the dynamic properties of a beam (primary system) during its passage, thus resulting in a time-dependent eigenvalue problem. Additional complications arise when an SDOF (secondary system) is attached to a beam for absorbing vibratory motion. The mathematical description of this coupled system is best handled by using energy considerations, while a solution is achieved by introducing generalized coordinates and using the Laplace transform with respect to time. Numerical results confirm vibratory energy absorption from the primary to the secondary system, despite the fact that the latter system lacks a dedicated damper and material damping is provided by the spring element only. Furthermore, these results are validated against experimental evidence using a simply supported model beam with hooks at the center span where the SDOF mechanism is attached. This way, it was possible to measure the beam’s response in the absence and then in the presence of the TMD, as its span was traversed by a heavy sliding mass. Given the fact that this simple, sub-optimal TMD helped to minimize the magnitude of vibrations exhibited by their supporting structure, a future goal for this research effort is to incorporate passive control within a structural health monitoring (SHM) environment so as to devise better ways to extend the useful service life of various categories of civil engineering infrastructure.

## 2. Optimum TMD Design

The performance of TMDs relies on a tuning process [4], in which their material parameters are optimized for one or several objectives. For this purpose, several formulations and numerical optimization methods have been developed. However, a given closed-form formulation may not be suitable for reaching a desired objective, as numerical optimization methods must be applied for different systems. For this reason, optimum TMD parameters such as fundamental period and damping ratio can also be estimated by using machine learning techniques [19]. Optimum expressions for the frequency $f_{opt}$ and damping ratio $\xi_{opt}$ for TMD design were proposed in [13] for harmonic motions and on the assumption that the primary structure is modeled as an SDOF system, followed by results derived in [14] for random vibrations. Furthermore, Ref. [20] examined optimum parameters of TMD, as gauged by their effectiveness in reducing accelerations and displacements caused by different earthquake excitations, on both SDOF and MDOF structures containing various numbers of TMD. Finally, Ref. [21] used an optimization method labeled ‘particle swarm’ to derive a set of TMD optimal values. All these results are reproduced in Table 1 and were further evaluated in [19], which also presented three new simplified equations for the optimal TMD frequency and damping ratio that were developed by using curve fitting of artificial neural network model results. In this last publication, the performance of the optimum TMD used in ten-story and forty-story frames was numerically evaluated by time history analyses for a set of twenty-two recorded seismic motions.

In essence, sufficiently realistic results cannot be obtained through classical calculations of the most suitable TMD parameters, since many problem variables are at work: (i) the type and number of TMD(s) used, (ii) the type of primary structure to which the TMD(s) are attached, (iii) the type and number of excitation(s), and (iv) optimization criteria, which can either address kinematic response minimization or the minimization of energies in the combined structural system. For more accurate results, metaheuristic methods have been employed for the optimization of various types of TMDs that have been developed, and this includes techniques such as ‘colony optimization’, ‘particle swarm’ optimization, ‘harmony search’ algorithms, genetic algorithms, gravitational algorithms, etc.; see [19] for a review. Information regarding optimum TMD design can be found in Table 1, where μ is the mass ratio between the TMD and the primary SDOF structure, which has a natural frequency $\omega_{1}$ and material damping ratio $\xi_{1}$, while the optimized frequency, mass and damping coefficient of the TMD are ω, m and c, respectively. Ref. [22] discusses the problem of balancing the reduction in the structural response with the amplitude of the TMD stroke, an issue that invariably leads to semi-active TMD design.

## 3. Coupled Primary–Secondary System with a Moving Heavy Mass

**Figure 1:**
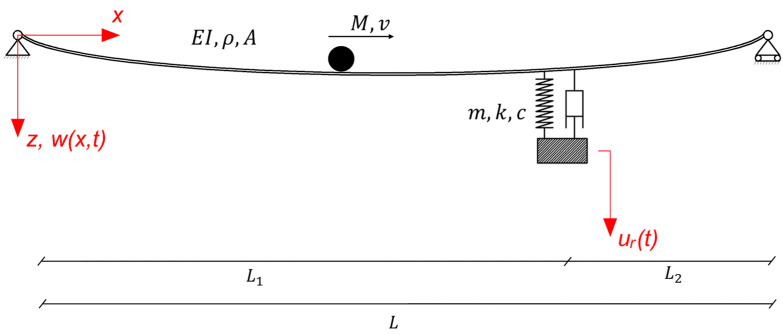
Simply supported Bernoulli–Euler beam with an attached SDOF system acting as the TMD and a moving point mass traversing the beam’s length.

As shown in Figure 1, a point mass M traverses a simply supported Bernoulli–Euler beam of length L with speed v, to which an SDOF system acting as a TMD is attached at location $x=L_{1}.$ As usual, EI,A,ρ are the flexural rigidity, the cross-section area and the mass density of the beam, while m,k,c are the lumped mass, spring constant and damping coefficient of the attached TMD. Furthermore, $w\left(x,t\right)$ is the transverse displacement of the beam and u(t) is the displacement of the mass of the TMD.

In order to identify the generalized coordinates of the coupled system, all displacements must be written with respect to the equilibrium position of both the beam and TMD; see Figure 1. By using the first two generalized coordinates $q_{i}$, we have $w\left(x,t\right)=\Phi_{i}\left(x\right)q_{i}\left(t\right),i=1,2$, where $\Phi_{i}$ are the eigenfunctions. The following position vectors are defined for the beam, the TMD and the moving mass; see Appendix A:

(1) $${\vec{r}}_{B}=\left(0,0,\Phi_{i}\left(x\right)q_{i}\left(t\right)\right),\;{\vec{r}}_{T}=\left(0,0,\Phi_{i}\left(L_{1}\right)q_{i}\left(t\right)+u_{r}\left(t\right)\right),\;{\vec{r}}_{M}=\left(vt,0,\Phi_{i}\left(vt\right)q_{i}\left(t\right)+r\left(x\left(t\right)\right)\right)$$

where $u_{r}(t)=u(t)-w\left(L_{1},t\right)$ is the relative displacement of the TMD with respect to the beam’s elastic curve, while $r\left(x\left(t\right)\right)$ is the roughness of the upper flange of the beam across which the point mass moves.

### 3.1. Potential Energy of the Coupled Structural System

The TMD is an SDOF system, and its potential energy about the static equilibrium position is simply $U_{T}=(1/2)ku_{r}^{2}$. Regarding the Bernoulli–Euler beam and considering flexural behavior, the potential energy is

(2) $$U_{B}=\frac{1}{2}\int_{\Omega }^{\;}\sigma_{xx}\varepsilon_{xx}\;d\Omega =\frac{1}{2}EI\int_{0}^{L}{\left(w^{\prime \prime }\right)}^{2}\;dx=\frac{1}{2}EI\int_{0}^{L}{\left(\Phi_{i}^{\prime \prime }(x)q_{i}(t)\right)}^{2}\;dx=\frac{1}{2}k_{ij}^{*}q_{i}q_{j}$$

where $k_{ij}^{*}=EI\int_{x=0}^{x=L}\Phi_{i}^{\prime \prime }\left(x\right)\Phi_{j}^{\prime \prime }\left(x\right)dx$ is the generalized stiffness of the beam and primes (′) indicate spatial derivatives. Given the orthogonality of the eigenfunctions and the fact that the second spatial derivative of an eigenfunction is $\Phi_{i}^{\prime \prime }\left(x\right)=-\kappa_{i}^{2}\Phi_{i}\left(x\right)$, where $\kappa_{i}$ is the wave number, we have $k_{ij}^{*}=EI\kappa_{i}^{2}\kappa_{j}^{2}\int_{x=0}^{x=L}\Phi_{i}\left(x\right)\Phi_{j}\left(x\right)dx=\omega_{i}^{2}\delta_{ij}$, so that finally, the potential energy of the beam is simply $U_{B}=(1/2)\omega_{i}^{2}q_{i}^{2}$, with $\omega_{i}$ being the eigenfrequencies.

### 3.2. Potential Energy of the Moving Mass

The potential energy of the point mass M as it moves along the top surface of the beam depends on the initial (in) and final (fi) transverse displacement as $U_{M}=U_{in}-U_{fi}=0-Mg\{\Phi_{i}\left(vt\right)q_{i}\left(t\right)+r(x,t)\}$, with g being the acceleration of gravity.

Adding all three components gives the total potential energy of the structural system as follows:

(3) $$U\left(q\right)=(1/2)ku_{r}^{2}+(1/2)k_{ij}^{*}q_{i}q_{j}-Mg\left\{\Phi_{i}\left(vt\right)q_{i}\left(t\right)+r\left(x\left(t\right)\right)\right\}$$

which depends on the kinematic vector $q=\left(q_{i},u_{r}\right),i=1,2$.

### 3.3. Kinetic Energy of the Coupled Structural System

The total time derivative of a material point with position vector $\vec{r}\left(t,q_{i},u\right),i=1,2$, is the velocity, i.e.,

(4) $$\vec{\nu }=d\vec{r}/dt=\partial \vec{r}/\partial t+(\partial \vec{r}/\partial q_{i}){\dot{q}}_{i}+(\partial \vec{r}/\partial u_{r}){\dot{u}}_{r}$$

where the overdot (.) indicates a time derivative. Taking into account the position vectors defined in Equation (1), the velocity of any point on the beam is ${\vec{\nu }}_{B}=\left(0,0,\Phi_{i}\left(x\right){\dot{q}}_{i}\left(t\right)\right)$, that of the TMD is ${\vec{\nu }}_{T}=\left(0,0,\Phi_{i}\left(L_{1}\right){\dot{q}}_{i}\left(t\right)+{\dot{u}}_{r}\left(t\right)\right)$ and that of the moving mass is ${\vec{\nu }}_{Μ}=\left(v,0,\Phi_{i}^{\prime }\left(vt\right)vq_{i}\left(t\right)+\Phi_{i}\left(vt\right){\dot{q}}_{i}\left(t\right)+r^{\prime }\left(x\left(t\right)\right)v\right)$. Note that in deriving the velocities, use was made of the partial derivative $\partial \vec{r}/\partial t$. The kinetic energy of a mechanical system is given by the generic formula $Τ=(1/2)m\nu^{2}$, where

(5) $$\nu^{2}=\vec{\nu }\vec{\nu }={\left(\frac{\partial \vec{r}}{\partial t}\right)}^{2}+2\frac{\partial \vec{r}}{\partial t}\left(\frac{\partial \vec{r}}{\partial q_{i}}{\dot{q}}_{i}+\frac{\partial \vec{r}}{\partial u_{r}}{\dot{u}}_{r}\right)+{\left(\frac{\partial \vec{r}}{\partial q_{i}}{\dot{q}}_{i}+\frac{\partial \vec{r}}{\partial u_{r}}{\dot{u}}_{r}\right)}^{2}$$

Therefore, summing the kinetic energy of the constituent parts gives the total kinetic energy as

(6) $$Τ=\left(1/2\right)m_{ij}^{*}{\dot{q}}_{i}{\dot{q}}_{j}+\left(1/2\right)\rho AL{\left(\Phi_{i}\left(L_{1}\right){\dot{q}}_{i}\left(t\right)+{\dot{u}}_{r}\left(t\right)\right)}^{2}+\;\left(1/2\right)Mv^{2}+(1/2)M{\left(\Phi_{i}^{\prime }\left(vt\right)\;v\;q_{i}\left(t\right)+\Phi_{i}\left(vt\right){\dot{q}}_{i}\left(t\right)+r^{\prime }\left(x\left(t\right)\right)\;v\right)}^{2}$$

In the above, the generalized mass is defined as $m_{ij}^{*}=\rho A\int_{x=0}^{x=L}\Phi_{i}\left(x\right)\Phi_{j}\left(x\right)dx$, which is simply equal to $m_{ij}^{*}=\delta_{ij}$ (the Kronecker delta) when taking into account the orthogonality property of the eigenfunctions.

## 4. Equations of Motion of the Combined Structural System

By substituting the energy terms in Lagrange’s equation, see Equation (A4) in Appendix A, and keeping in mind that the dependent variables of the problem are $q=\left(q_{1},{q_{2},u}_{r}\right)$, we recover the standard form of the equations of motion where the inertia, damping and restoring forces in the structural system balance the externally applied loads F as follows:

(7) $$M\left(t\right)\ddot{q}\left(t\right)+C\left(t\right)\dot{q}\left(t\right)+Κ\left(t\right)q\left(t\right)=F\left(t\right)$$

Note that the mass M, damping C and stiffness K matrices are all time-dependent, as is the forcing function F, and are listed below:

(8) $$M\left(t\right)=\left[\begin{array}{ccc} m_{11}^{*} & m_{12}^{*} & 0\\ m_{21}^{*} & m_{22}^{*} & 0\\ 0 & 0 & 1 \end{array}\right]+\left[\begin{array}{ccc} Μ\Phi_{1}\left(vt\right)\Phi_{1}\left(vt\right) & {Μ\Phi }_{1}\left(vt\right)\Phi_{2}\left(vt\right) & 0\\ Μ\Phi_{2}\left(vt\right)\Phi_{1}\left(vt\right) & Μ\Phi_{2}\left(vt\right)\Phi_{2}\left(vt\right) & 0\\ 0 & 0 & 0 \end{array}\right]\;+\left[\begin{array}{ccc} m\Phi_{1}\left(L_{1}\right)\Phi_{1}\left(L_{1}\right) & m\Phi_{1}\left(L_{1}\right)\Phi_{2}\left(L_{1}\right) & {m\Phi }_{1}\left(L_{1}\right)\\ {m\Phi }_{2}\left(L_{1}\right)\Phi_{1}\left(L_{1}\right) & m\Phi_{2}\left(L_{1}\right)\Phi_{2}\left(L_{1}\right) & {m\Phi }_{2}\left(L_{1}\right)\\ \Phi_{1}\left(L_{1}\right) & \Phi_{2}\left(L_{1}\right) & 0 \end{array}\right]\;C\left(t\right)=\left[\begin{array}{ccc} c_{11}^{*} & c_{12}^{*} & 0\\ c_{21}^{*} & c_{22}^{*} & 0\\ 0 & 0 & 2\omega \xi \end{array}\right]+\left[\begin{array}{ccc} 2Mv\Phi_{1}\left(vt\right)\Phi_{1}^{\prime }\left(vt\right) & {2Mv\Phi }_{1}\left(vt\right)\Phi_{2}^{\prime }\left(vt\right) & 0\\ {2Mv\Phi }_{2}\left(vt\right)\Phi_{1}^{\prime }\left(vt\right) & {2Mv\Phi }_{2}\left(vt\right)\Phi_{2}^{\prime }\left(vt\right) & 0\\ 0 & 0 & 0 \end{array}\right]\;Κ\left(t\right)=\left[\begin{array}{ccc} k_{11}^{*} & k_{12}^{*} & 0\\ k_{21}^{*} & k_{22}^{*} & 0\\ 0 & 0 & \omega^{2} \end{array}\right]+\left[\begin{array}{ccc} Mv^{2}\Phi_{1}\left(vt\right)\Phi_{1}^{\prime \prime }\left(vt\right) & {Mv^{2}\Phi }_{1}\left(vt\right)\Phi_{2}^{\prime \prime }\left(vt\right) & 0\\ Mv^{2}\Phi_{2}\left(vt\right)\Phi_{1}^{\prime \prime }\left(vt\right) & Mv^{2}\Phi_{2}\left(vt\right)\Phi_{2}^{\prime \prime }\left(vt\right) & 0\\ 0 & 0 & 0 \end{array}\right]\;F\left(t\right)=\left\{\begin{array}{c} \left(Mg-Mv^{2}\;r^{\prime \prime }\left(x\left(t\right)\right)\right)\Phi_{1}\left(vt\right)\\ \left(Mg-Mv^{2}\;r^{\prime \prime }\left(x\left(t\right)\right)\right)\Phi_{2}\left(vt\right)\\ 0 \end{array}\right\}$$

We know that the generalized masses, stiffnesses and dampers, respectively, are $m_{ij}^{*}$ = $\delta_{ij}$, $k_{ij}^{*}=\omega_{i}^{2}\delta_{ij}$ and $c_{ij}^{*}=2\omega_{i}\xi_{i}\rho A\delta_{ij}$, with $\omega_{i}$ being the eigenfrequencies of the beam in the absence of both TMD and moving mass, while $\xi_{i}$ are the damping coefficients associated with the first two eigenmodes. Obviously, it is quite simple to add to the above equation more TMD systems.

### Free Vibrations

As the point mass moves across the span of the bridge, it is possible to define a critical velocity as $v_{cr}=(\pi /L)\sqrt{EI/\left(\rho A\right)}$, past which the maximum transvers displacement will occur in the free vibration regime. For the simply supported beam, $v_{cr}=\omega_{1}$, meaning that it coincides with the first eigenfrequency. However, if the moving mass is heavy as compared to the weight of the beam, the eigenvalue problem becomes time-dependent during the passage of the mass, which implies that all eigenfrequencies $\omega_{i},i=1,2,\ldots$ are no longer constant but time-dependent. Thus, it becomes important to focus on the free vibrations of the combined beam-TMD structural system. Thus, F=0 in the equation of motion, Equation (8), leaving the following terms:

(9) $$\begin{array}{c} M\left(t\right)=\left[\begin{array}{ccc} m_{11}^{*} & m_{12}^{*} & 0\\ m_{21}^{*} & m_{22}^{*} & 0\\ 0 & 0 & 1 \end{array}\right]+\left[\begin{array}{ccc} m\Phi_{1}\left(L_{1}\right)\Phi_{1}\left(L_{1}\right) & m\Phi_{1}\left(L_{1}\right)\Phi_{2}\left(L_{1}\right) & m\Phi_{1}\left(L_{1}\right)\\ m\Phi_{2}\left(L_{1}\right)\Phi_{1}\left(L_{1}\right) & m\Phi_{2}\left(L_{1}\right)\Phi_{2}\left(L_{1}\right) & m\Phi_{2}\left(L_{1}\right)\\ \Phi_{1}\left(L_{1}\right) & \Phi_{2}\left(L_{1}\right) & 0 \end{array}\right]\\ C\left(t\right)=\left[\begin{array}{ccc} c_{11}^{*} & c_{12}^{*} & 0\\ c_{21}^{*} & c_{22}^{*} & 0\\ 0 & 0 & 2\omega \xi \end{array}\right]\\ K\left(t\right)=\left[\begin{array}{ccc} k_{11}^{*} & k_{12}^{*} & 0\\ k_{21}^{*} & k_{22}^{*} & 0\\ 0 & 0 & \omega^{2} \end{array}\right]\\ F\left(t\right)=\left\{\begin{array}{c} \left(Mg-Mv^{2}r^{\prime \prime }\left(x\left(t\right)\right)\right)\Phi_{1}\left(vt\right)\\ \left(Mg-Mv^{2}r^{\prime \prime }\left(x\left(t\right)\right)\right)\Phi_{2}\left(vt\right)\\ 0 \end{array}\right\} \end{array}$$

The initial conditions are the vibrations imparted on the beam at the instant the moving load leaves the span of the beam.

## 5. Solution Strategy Using the Laplace Transform

Since the matrices in the equation of motion are time-dependent, we will employ the Laplace transform defined for a function of time f(t) as follows [23]:

(10) $$L\left\{f\left(t\right)\right\}=F\left(s\right)=\int_{0}^{\infty }f\left(t\right)\exp\left(-st\right)dt,\;L^{-1}\left\{F\left(s\right)\right\}=\int_{c-i\infty }^{c+i\infty }F\left(s\right)\exp\left(st\right)ds$$

Note that t s is the Laplace transform parameter and the inversion integral is defined over the complex plane. The solution strategy is to discretize the time axis as $t_{n}=n\Delta t,n=1,2,3,\ldots ,N$ and transform the matrix equation of motion, Equation (8), in the Laplace domain sequentially for every time step increment nΔt. Once the problem has been solved, it is followed by the inverse Laplace transform based on Talbot’s algorithm [24] for returning values back to the time domain. It is assumed that the time step Δt is small enough for the system matrices and external force in Equation (8) to remain constant over a time interval. Furthermore, the total time of interest for the beam’s motion is $t_{b}=L/v=N\Delta t$. The result is a system of 3×3 matrix algebraic equations which are Laplace-transformed, which makes them parametric in the Laplace transform variable s:

(11) $$\left(Μ\left(n\Delta t\right)s^{2}+C\left(n\Delta t\right)s+K\left(n\Delta t\right)\right)Q\left(s\right)=\;\;s\;\left(Μ\left(n\Delta t\right)q\left(0\right)\right)+\left(Μ\left(n\Delta t\right)\dot{q}\left(0\right)+C\left(n\Delta t\right)q\left(0\right)+F\left(n\Delta t\right)/s\right)$$

We know that the vector of the unknown Laplace transformed kinematic variables is $Q^{T}\left(s\right)=\left\lfloor \begin{array}{ccc} Q_{1}\left(s\right) & Q_{2}\left(s\right) & U_{r}\left(s\right) \end{array}\right\rfloor$, while the initial conditions for the generalized displacements and velocities are $q^{T}\left(0\right)=\left\lfloor \begin{array}{ccc} q_{1}(0) & q_{2}(0) & u_{r}(0) \end{array}\right\rfloor$ and ${\dot{q}}^{T}\left(0\right)=\left\lfloor \begin{array}{ccc} {\dot{q}}_{1}(0) & {\dot{q}}_{2}(0) & {\dot{u}}_{r}(0) \end{array}\right\rfloor$, respectively, and are the final conditions from the immediately previous time interval $t_{i-1}=\left(n-1\right)\Delta t$. Also, superscript **T** stands for the transpose operator. Following the solution of Equation (11) for $Q\left(s\right)$, the inverse Laplace transformation is applied numerically (see Appendix B) to recover the vector of the kinematic variables in the time domain as q(Δt). Finally, the procedure is repeated until the entire time axis has been swept. Note that all computations were carried out in the Python [25] software environment.

## 6. Numerical Implementation

The methodology developed above is applied to the case of a simply supported HEB 100 steel beam under heavy point mass sliding with constant velocity v over the span length L. Since this analytical model corresponds to an actual experimental setup [26], all values reported in Table 2 were also measured in the laboratory for consistency. In what follows, the dynamic response of the beam with the sliding mass was first computed both in the absence and then in the presence of a TMD, which is a simple SDOF system comprising a mass and a spring. Finally, the roughness function r(x) describing the passage of the moving mass over the beams top flange was measured by a quasi-static passage of the mass and is a nearly white noise random function of space with a small amplitude, common in all analyses.

### Analytical Investigation

As will be discussed in conjunction with the experimental measurements, the presence of the TMD was investigated by computing the acceleration response of the structural system to the moving mass at three stations, namely at $x{=L}_{1}=L/2-0.212\;m=2.70\;m$ and at x=3L/4=4.37 m on the span measured from the right end, and again at x=2.70 m but on the TMD itself. The computations yielded both time histories and frequency spectra for the accelerations, which will be shown in the next section along with the experimental measurements.

**Figure 2:**
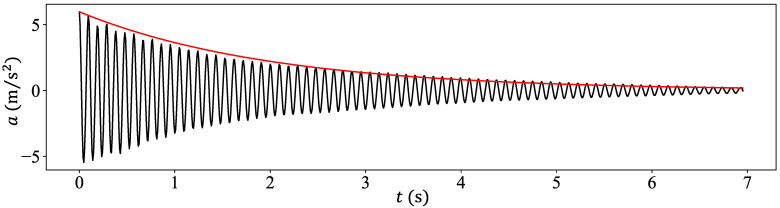
The free vibration accelerogram of the SDOF system following an impact hammer excitation with the red curve showing the exponential decay of the amplitude.

It should be noted that the TMD is an SDOF system that was manufactured in the laboratory and consisted of a metallic spring supporting two metallic discs. The mass ratio between the SDOF system (secondary system) and the beam (primary system) was μ=m/ρAL=0.0278/0.116=0.24. Next, the metallic spring had a stiffness k such that the frequency ratio between the TMD and the beam dominant frequencies was $\omega_{1}/\omega =9.80/10.54=0.93$. This value is close enough to unity, which is the key parameter in designing a TMD [4]. Note that damping in the TMD is provided by viscous damping inherent in the metallic spring. This was evaluated by striking the TMD with an impact hammer that induced a free vibration regime, followed by use of the logarithmic decrement method to measure the viscous damping ratio ξ; see Figure 2.

Table 3 compares the optimal TMD mechanical parameter values suggested by [13] versus those actually used here, where it is observed that in our case, damping is minimal, leading to a sub-optimal TMD design. The benefit is that it is not necessary to manufacture and attach a damper to the original SDOF system, i.e., a simple and economic design is used.

## 7. Experimental Investigation

### 7.1. Description of the Experiment

**Figure 3:**
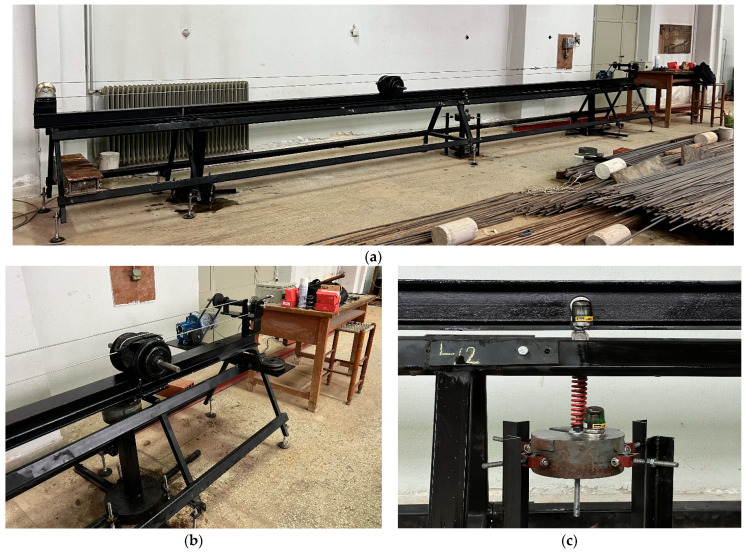
Experimental setup showing the (**a**) simply supported beam; (**b**) the moving mass with the electric motor driving it through a wire, plus the base station at the right end; and (**c**) the TMD with the four metallic guides to ensure vertical motion only.

The experimental setup is shown in Figure 3, where a 5.83m, HEB 100 steel beam segment was simply supported by two tripods, with 1 m approaches to the left and right of the supports. The moving mass could reach up to 50 kg and slid across the upper beam flange with a maximum speed of up to 50 cm/s through a cable pulled by an electric motor–gear box configuration. The TMD was a spring-mass setup hanging vertically at the point of contact with the lower flange 2.70 m from the right support. It was constrained by four guides with ball bearings to move in the vertical direction only, thus avoiding any lateral motion. The instrumentation [27] comprised three wireless sensors (i.e., accelerometers measuring motion in the three principal directions) placed at stations $x=L_{1}=2.70\;m,x=3L/4=4.37\;m$, as measured from the right support, with the third placed directly on the TMD. The sensors transmitted signals to a nearby base receiver, which in turn was connected to a laptop computer with built-in software for real-time processing of the incoming signals in both time and frequency domains; see Table 4 for details. These signals corresponded to the transverse accelerations of the beam at the two aforementioned stations plus the vertical motion of the TMD mass.

### 7.2. Comparison between Analytical and Experimental Results

**Figure 4:**
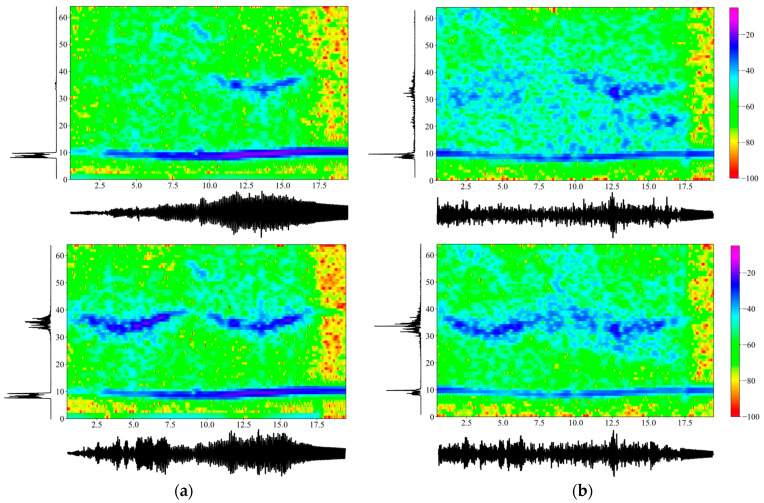
Comparison between the (**a**) numerically obtained and (**b**) the experimentally measured acceleration time histories ($m/s^{2})$, the PSD of the Fourier transforms ${\left((m/s^{2}\right)}^{2}/Hz$) and the spectrograms (dB/Hz) in the absence of the TMD and for an intermediate moving mass case of M=27 kg,v=33 cm/s. Note: Upper graphs are at location $L_{1}=2.70\;m$; bottom graphs are at location 3L/4=4.37 m.

The comparison study was carried out for a number of cases to establish agreement between the analytical and the experimental results. Firstly, Figure 4 plots numerically obtained accelerations in the left column and experimentally measured ones in the right column, both in the absence of the TMD. The scenario considered was the intermediate case of a mass M=27 kg sliding with a velocity of v=33 cm/s. Specifically, acceleration time histories are plotted horizontally, their power spectral densities are plotted vertically and the central part depicts spectrograms [15], i.e., time–frequency plots of the accelerations. In our case, spectrograms were derived from the Short Time Fourier Transform (SHFT), which is a sequence of Fourier transforms of a windowed signal that provides the time-localized frequency information for cases in which frequency components of a signal vary over time. More specifically, a Hanning window with a 128 data-point width was used, while two consecutive windows overlapped 120 data-points.

**Figure 5:**
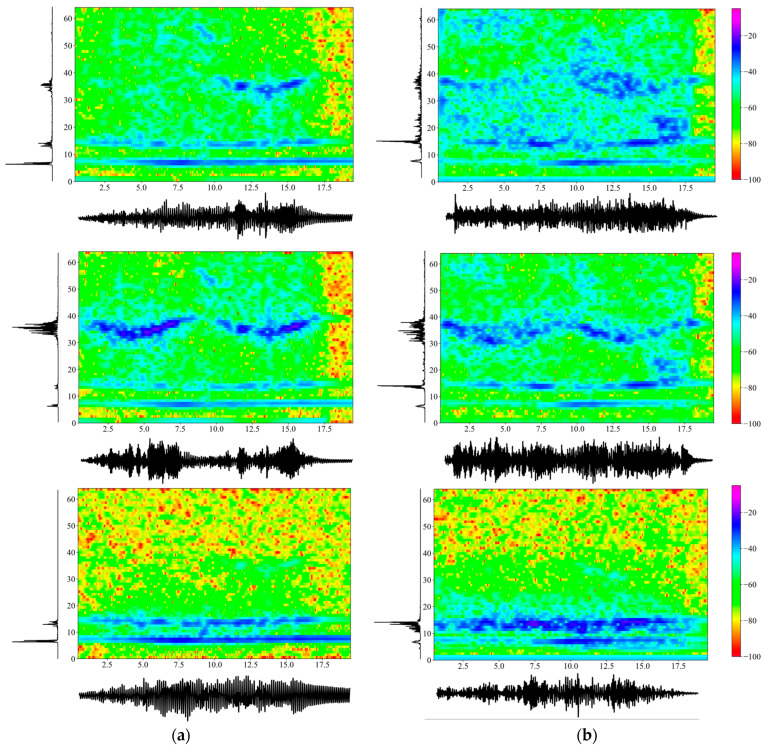
Comparison between the (**a**) numerically obtained and (**b**) the experimentally measured acceleration time histories ($m/s^{2})$, the PSD of the Fourier transforms, $\left(({m/s^{2})}^{2}/Hz\right)$ and the spectrograms (dB/Hz) in the presence of the TMD and for an intermediate moving mass case of M=27 kg,v=33 cm/s. Note: Upper graphs are at location $L_{1}=2.70\;m$; middle graphs are at location 3L/4=4.37 m; bottom graphs are at the TMD location. Schemes follow the same formatting.

The time axis ran to 20 s, which covered the transit time of the mass plus a free vibration regime of about 3 s. Note that power spectral density (PSD) function is an energy measure of the Fourier transform F(f) of time function f(t) and at a given frequency point $f_{i}$, defined as $PSD(fi)=F(fi)\overset{-}{F}(fi)/N$, where N is the number of sampling points and the overbar (–) denotes the complex conjugate. The frequency axis went up to 64 Hz, and the transient accelerations had a maximum amplitude of $0.03\;m/s^{2}$, with the same scale used for all graphs. Next, Figure 5 depicts the same information as Figure 4, but in the presence of the TMD. The good agreement between numerical and experimental results should be noted, especially when the uncertainties in the experiment (e.g., the roughness of the upper flange of the beam, the initial acceleration of the sliding mass, the transition from the guide to the span, etc.) are taken into account.

Comparing Figure 4 and Figure 5, the acceleration amplitude is reduced by a small amount across time when the TMD is attached to the beam close to center span at $x=L_{1}$. The power spectral density of the acceleration records shows a split of the first eigenfrequency of 9.80 Hz into values of around 7 Hz and 14 Hz. Note that the splitting of a beam’s fundamental frequency because of the presence of a TMD is a phenomenon well established in the literature [4]. However, this is not true at higher frequencies, e.g., around 39 Hz, where the second natural frequency of the beam lies, since this region is beyond the influence of the TMD. All these observations are true for both numerical and experimental results. Next, an energy metric of the time domain signal is simply the sum of the squares of the accelerations at each time step, computed here at station $L_{1}=2.70\;m$ as 8.15 in the absence and as 7.94 in the presence of the TMD. This particular metric derived from the experimental measurements clearly shows energy absorption by the TMD, which is further confirmed at station $L_{1}=3L/4$, where the respective metrics are equal to 7.34 and 5.80.

In addition, Figure 5 shows the computed transient acceleration recorded at the TMD, plus its PSD when this record is transformed to the frequency domain. Obviously, the TMD also absorbs vibratory energy, and its acceleration response is comparable in magnitude to that of the beam. Note that what Figure 5 shows is total accelerations, i.e., $\ddot{u}(t)={\ddot{u}}_{r}(t)+\ddot{w}(L/2,t)$. As before, the PSD of the TMD clearly shows a peak in the low frequency band where both the beam’s first natural frequency and the TMD frequency lie, i.e., at around f=10 Hz. The smaller peak at around 15 Hz is of minor consequence and represents the influence of the primary structure (the beam) on the secondary one (the TMD). Similar results were obtained for various sliding mass and traverse velocity combinations.

In addition, both Figure 4 and Figure 5 plot spectrograms in the combined time–frequency domains at two beam stations, i.e., at $x=L_{1},x=3L/4$, due to the passage of the traveling mass. The purpose is to compare computed and experimental results in the absence and then the presence of the TMD, with a spectrogram given for the vertical acceleration recorded on the TMD itself. In the first case, the trace of the first two eigenfrequencies of the beam is clearly discernable, meaning that their variation over time as the mass moves across the beam is visible. In the latter case, the presence of the TMD still preserves this characteristic, although it is not as clear as before. Furthermore, what these spectrograms show is the split in the fundamental frequency of the combined primary–secondary system over time when the TMD is present, a feature that is crucial in setting up machine learning algorithms for identifying the presence of secondary systems.

### 7.3. TMD Performance

**Figure 6:**
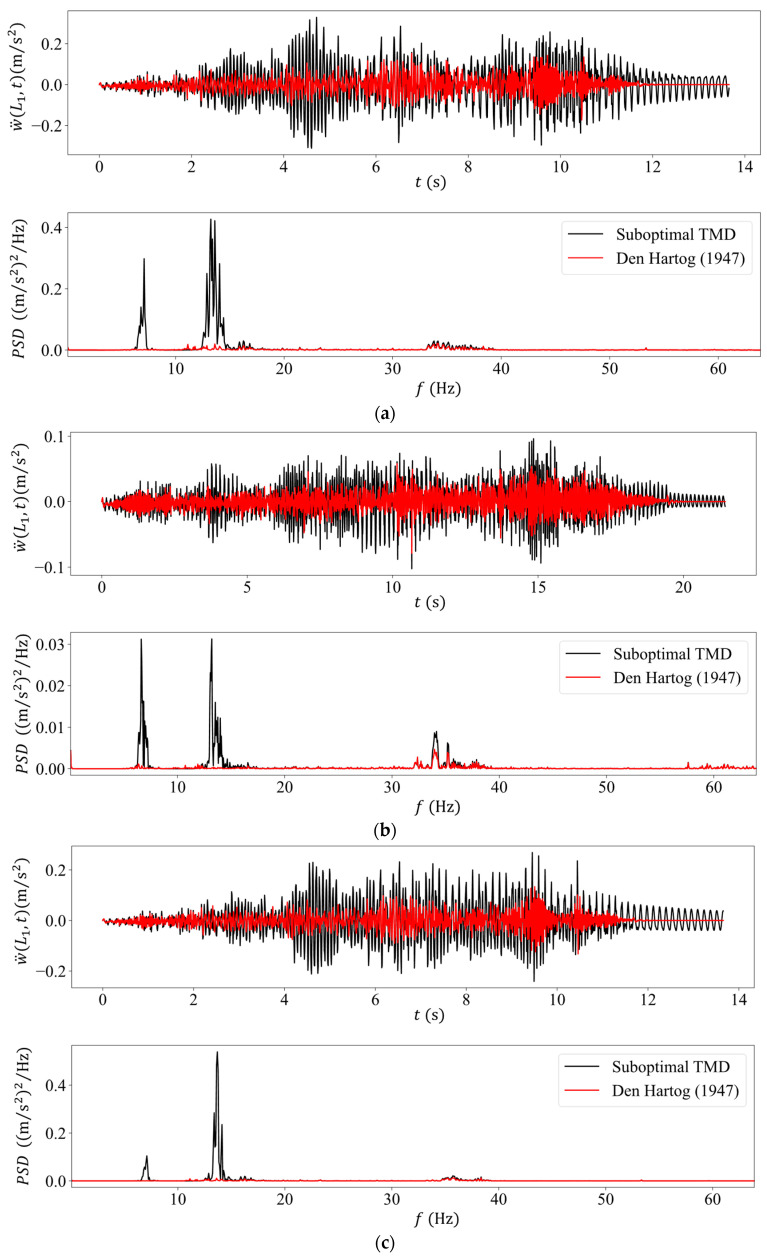

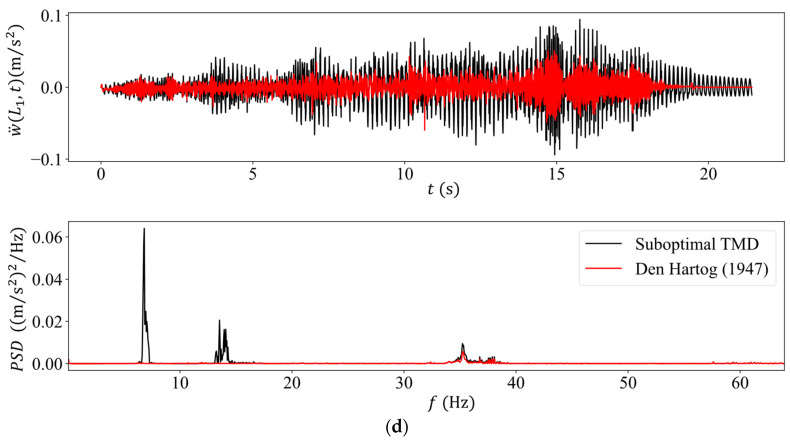
Numerically computed acceleration time histories (top graph) and their PSDs (bottom graph) for the sub-optimal TMD used in the experiments (black color) versus the optimum TMD as defined by Den Hartog [13] (red color) for the intermediate sliding mass case of (**a**) M=27 kg,v=50 cm/s, (**b**) M=27 kg,v=30 cm/s, (**c**) M=18 kg,v=50 cm/s and (**d**) M=18 kg,v=30 cm/s.

Two basic sets of results are presented in Figure 6 to elucidate the role of the present sub-optimal TMD in controlling the beam vibrations due to the passage of a heavy mass, as compared to a fictitious optimal design [13] that would include a damper in addition to the mass and spring design. Thus, Figure 6 plots the acceleration time histories and the PSDs of their Fourier transform at the key TMD location $x=L_{1}$ near the center of the span for these two designs. Four cases are examined, namely the original mass value of M=27 kg traveling at two velocities, the reference value of v=33 cm/s and a higher velocity of v=50 cm/s, plus a smaller mass of M=18 kg traveling at both these velocities. At first, we observe in the transient acceleration plots that the performance of the suboptimal TMD is not as effective as that of the optimal TMD, but this difference in performance is not great, as there are time intervals where the former TMD performs just as well as the latter one. However, the optimal TMD design becomes more effective towards the end of the passage of the sliding mass when the free vibration regime sets in.

What is interesting to note in the PSD spectra is that in the case of the sub-optimal TMD, the first (original) eigenfrequency has split in two, while for the case of the optimal TMD (Den Hartog), it has completely disappeared. This implies that the beam’s response in the presence of the optimal TMD is primarily synthesized from high-frequency components.

Next, the range of the sliding mass magnitude plays a lesser role as compared to its velocity. More specifically, both TMD designs are more effective at the lower velocity of 30 cm/s as compared to the higher velocity of 50 cm/s, irrespective of the sliding mass magnitude. This holds true for both the magnitude of the transient accelerations as well as that of the PSD spectra. The controlling factor here is that the faster traverse velocity produces higher-frequency beam vibrations as compared to the lower velocity, while the TMDs are designed to be effective around the first beam eigenfrequency region.

### 7.4. Energy Flow in the TMD

**Figure 7:**
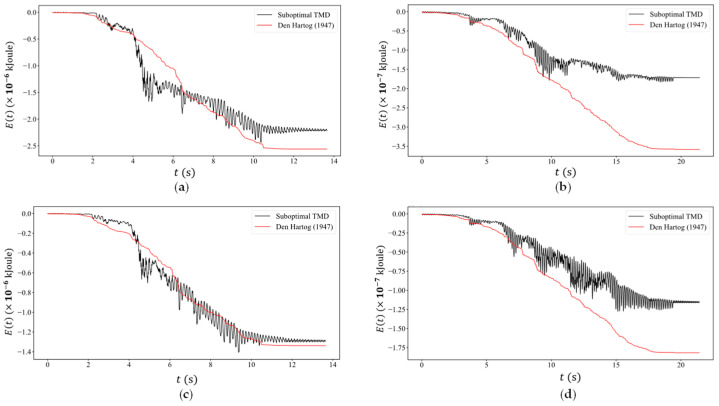
Energy accumulation over time in the sub-optimal TMD used here (black color) versus the optimum TMD as defined by Den Hartog [13] (red color) for the sliding mass cases of (**a**) M=27 kg,v=50 cm/s, (**b**) M=27 kg,v=30 cm/s,(c)M=18 kg,v=50 cm/s and (**d**) M=18 kg,v=30 cm/s.

Finally, an important parameter to consider is the energy flow over time in the TMD, which is quantified by the energy measure E(t) given in Figure 7. This energy is dependent on the force $F_{TMD}(t)$ which develops at the point of attachment between TMD and the supporting beam, as well as on the beam velocity as the heavy mass traverses the beam:

(12) $$E\left(t\right)=\int_{0}^{t}F_{TMD}\left(\tau \right)\;\dot{w}\left(L_{1},\;\tau \right)d\tau$$

This contact force in turn is evaluated from the equation of motion of the TMD as follows:

(13) $$m{\ddot{u}}_{r}+c{\dot{u}}_{r}+ku_{r}=-m\ddot{w}\left(L_{1},t\right)\overset{\;}{\Rightarrow }F_{TMD}\left(t\right)=c{\dot{u}}_{r}(t)+ku_{r}(t)=-m\ddot{w}\left(L_{1},t\right)-m{\ddot{u}}_{r}(t)$$

What is important to observe in Figure 7, which contrasts the present suboptimal TMD design with a fictitious optimal one, is that both designs absorb similar amounts of energy as the traveling mass approaches the center of the span. After that, the optimal TMD design becomes more effective by absorbing more vibratory energy, but this stops in the free vibration regime past 17.6 s. By this point, however, vibrations at the center span have started to decrease anyway. Note that the optimal TMD design shows a continuous energy absorption, while our sub-optimal TMD, which has minimal damping, cannot do so, and as a result, there is some flow of vibratory energy back to the beam. In terms of the sliding mass magnitude and velocity, we observe that the energy transfer in the suboptimal TMD closely follows that observed in the optimal TMD at the higher velocity of v=50 cm/s, and less so at the lower velocity of v=30 cm/s. This happens irrespective of the sliding mass magnitude and again demonstrates that the sliding mass velocity is a more critical parameter as compared to its mass. Finally, since the sub-optimal TMD absorbs less vibratory energy, it will be less impaired over time when multiple masses have traveled across the bridge’s span.

## 8. Discussion and Conclusions

Tuned mass dampers were first introduced nearly one hundred years ago in structures and have since been shown to be efficient, reliable and cost-effective systems for vibration control. The design of TMDs in civil engineering addresses two basic categories of loads, namely wind-induced pressures and seismically induced ground motions. However, a large, tuned mass is often required in TMD design to adjust the combined structural system dynamic characteristics and to reach pre-set performance targets, a process that might lead to secondary transient effects in the structure. The use of TMDs and other, similar SDOF or MDOF devices in mechanical engineering serves an additional function that has to do with energy harvesting. Specifically, the kinetic energy absorbed by such secondary systems during pronounced vibrations of beams and other structural elements to which they are attached can theoretically be stored and used for commercial purposes. Often, these structural elements are excited by external forces of high magnitude such as wind pressure and as a result operate in the nonlinear range, exhibiting regions of instability.

The novelty in the present work, which focuses on vibration control in bridges, is the combination of an analytical solution plus its experimental verification regarding the flexural displacements induced in a bridge due to a heavy mass traveling across its span when a sub-optimal TMD is attached to a fixed location. The purpose of this simple TMD that comprises only a mass and a spring is to ameliorate the induced flexural vibrations with the aim of prolonging the service life of the bridge. Note that the only damping available in this suboptimal TMD is that provided by the spring element. Next, it is known that heavy traveling masses modify the dynamic properties of the bridge during the traverse time, thus resulting in a time-dependent eigenvalue problem. Furthermore, the dynamic response of the bridge for the case described above depends on the speed of traverse and on the mass ratio between the traveling mass and the bridge.

In closing, determining the response of a TMD with pre-set mechanical properties placed at fixed locations along the span is not a trivial problem to analyze, and the literature does not give guidelines in the case of continuous dynamic systems supporting heavy moving mass loads. Thus, the first step in this direction was to analyze the dynamic response of a simply supported bridge deck modeled as a continuous mass distribution system (i.e., a waveguide) with a TMD placed about the center of its span to counter the effects of a heavy traveling mass. Furthermore, it was essential to conduct experiments to verify that a simple, economical and easy to install TMD was capable of absorbing vibrations. This was successfully demonstrated by the numerical analysis results, which in turn were validated by the experimental measurements, all showing good agreement for the structural configuration presented herein. Finally, there are a few more considerations that must be addressed in the future such as (i) optimization studies on different TMD configurations, (ii) application to multiple categories of dynamic loads such as wind pressure and ground-induced motions, (iii) the use of multiple TMD configurations and (iv) the consideration of multiple span bridges.

## Figures and Tables

**Table 1:** TMD frequency $f_{opt}$ and damping ratios $\xi_{opt}$ for optimal design.

Method	$f_{opt}=\omega /\omega_{1}$	$\xi_{opt}=c/2m\omega$
Den Hartog (1947) [13]	1/(1+μ)	$\sqrt{3\mu /8(1+\mu )}$
Warburton (1982) [14]	$\sqrt{1-\mu /2}/\left(1+\mu \right)$	$\sqrt{\mu (1-\mu /4)/4(1+\mu )(1-\mu /2)}$
Sadek et al. (1997) [20]	$\left[1-\xi_{1}\sqrt{\mu /\left(1+\mu \right)}\right]/\left(1+\mu \right)$	$\xi_{1}/\left(1+\mu \right)+\sqrt{\mu /\left(1+\mu \right)}$
Leung and Zhang (2009) [21]	$\sqrt{1-\mu /2}/\left(1+\mu \right)$ $+\left(20.23\sqrt{\mu }-37.94\mu -4.945\right)\sqrt{\mu }\xi_{1}$ $+\left(25.00\sqrt{\mu }-4.829\right)\sqrt{\mu }\xi_{1}^{2}$	$\sqrt{\mu (1-\mu /4)/4(1+\mu )(1-\mu /2)}-5.302\xi_{1}^{2}\mu$

**Table 2:** Combined primary–secondary system mechanical properties.

The Bernoulli–Euler Beam							
ρ $\left(tn/m^{3}\right)$	A $\left(m^{2}\right)$	E (GPa)	I $\left(m^{4}\right)$	$\xi_{1}$	$\xi_{2}$	ρAL (tn)	L (m)
7.65	$26\cdot 10^{-4}$	198.5	$450\cdot 10^{-8}$	0.0021	0.0084	0.116	5.83
**The moving mass reference properties and the TMD properties**							
Μ (tn)	v (m/s)	$\mathrm{TMD}\;\mathrm{location}\;L_{1}$ (m)		TMD mass m (tn)	TMD stiffness k (kN/m)		TMD damping ξ
0.027	0.33	2.70		0.0278	122.0		0.0075

**Table 3:** Optimal versus sub-optimal TMD mechanical properties for a fixed TMD mass m=27.8 kg.

Den Hartog (1947) [13] Optimal TMD Model			The TMD Used Here		
$f_{opt}(Hz)$	$\xi_{opt}$	$k_{opt}$ (kN/m)	f (Hz)	ξ	k (kN/m)
7.90	0.2692	68.53	10.54	0.0075	122.0

**Table 4:** Wireless sensor network specifications.

Wireless Triaxial Accelerometer Node G-Link-200		Wireless Sensor Data Aggregator WSDA-2000	
Measurement range	±8 g	Radio frequency transceiver carrier	License-free 2.405 to 2.480 GHz with 16 channels
Noise density	$25\;mg/\sqrt{Hz}$		
Resolution	20 bit		
Sampling ratio	128 Hz		
Forced vibration regime $t_{b}=L/v$	17.67 s		
Free vibration regime $t_{f}$	1.5 s		

**Figure A1:**
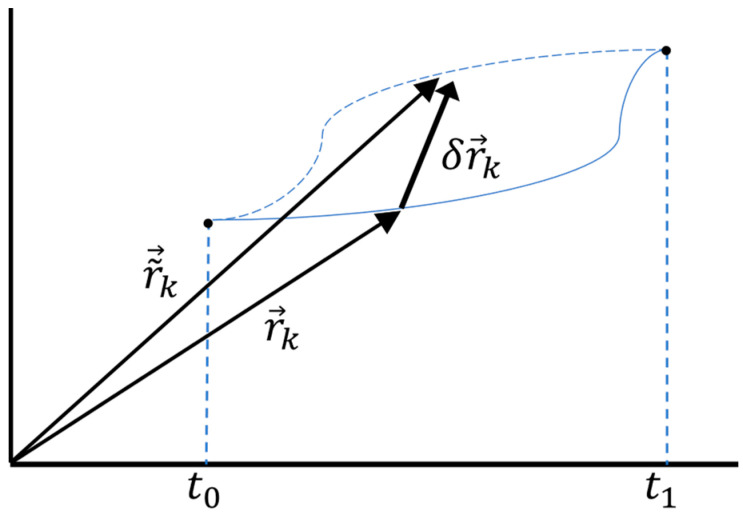
Position vector ${\vec{r}}_{k}$ for a point mass $m_{k}$ during time interval $\left[t_{0,}t_{1}\right]$ and its variation $\delta {\vec{r}}_{k}$.

## Data Availability

Data are contained within the article.

## Appendix A

In order to derive the equations of motion of a coupled primary–secondary system comprising a simply supported beam with an attached TMD under the influence of a traveling point mass, recourse is made to Lagrange’s equation [13]. Specifically, starting with the virtual work for a structural element with $m_{k}$ point masses at position vectors ${\vec{r}}_{k}$, acted upon by external forces ${\vec{P}}_{k}$ plus support reactions ${\vec{R}}_{k}$ as ${\vec{F}}_{k}={\vec{P}}_{k}+{\vec{R}}_{k}$, we find that $\delta W=\sum_{k=1}^{N}{(\vec{F}}_{k}-m_{k}{\vec{\ddot{r}}}_{k})\delta {\vec{r}}_{k}=0$. Note that δ is the variation symbol and overdots (·) indicate time derivatives. In the aforementioned statement, $\delta {\vec{r}}_{k}$ is the first variation of position vector ${\vec{r}}_{k}(q_{k})$, which is assumed to be a function of the generalized coordinates $q_{k}\left(t\right),\;k=1,2,3,\ldots$ which are related to the physical coordinates through the eigenfunctions of the dynamic system. The time interval of interest is $\left[t_{0,}t_{1}\right]$, so that the variation at the two end points is $\delta {\vec{r}}_{k}\left(t_{0}\right)=\delta {\vec{r}}_{k}\left(t_{1}\right)=0$; see Figure A1. Following a Taylor series expansion with respect to the relative position $\Delta {\vec{r}}_{k}={\vec{\tilde{r}}}_{k}-{\vec{r}}_{k}$ between a virtual position ${\vec{\tilde{r}}}_{k}$ and the actual one, we recover $\delta W=Q_{i}\delta q_{i}$, where $Q_{i}\;$ are the generalized forces corresponding to the generalized coordinates $q_{i}$. Next, we define a scalar potential function $U(q_{i})$, such that $Q_{i}=-\partial U/\partial q_{i}$. Thus, virtual work comprises the conservative forces, the inertia forces including the kinetic energy T, plus any non-conservative forces (subscript NC):

(A1) $$\delta W=-\delta U+\delta W_{NC}+\sum_{k=1}^{N}\left(-\frac{d}{dt}\left(m_{k}\;{\vec{\dot{r}}}_{k}\;\delta {\vec{r}}_{k}\right)\right)+\delta T=0$$

If the above equation is integrated over the time interval $\left[t_{0,}t_{1}\right]$, the work by the individual point masses $m_{k}$ is zero and the result is

(A2) $$\int_{t_{0}}^{t_{1}}\delta Wdt=\int_{t_{0}}^{t_{1}}\left(-\delta U+\delta W_{NC}+\delta T\right)dt=0$$

The work performed by the non-conservative forces can be further decomposed to that performed by the external forces and that due to proportional damping as $\delta W_{NC}=Q_{i}\;\delta q_{i}-c_{i}{\dot{q}}_{i}\;\delta q_{i}$, where $c_{i}$ is the damping coefficient corresponding to the i−th generalized coordinate. Thus, the virtual work statement is

(A3) $$\int_{t_{0}}^{t_{1}}\left(\delta T-\delta U+Q_{i}\;\delta q_{i}-c_{i}{\dot{q}}_{i}\;\delta q_{i}\right)dt=0$$

Next, the kinetic energy term $T(q_{i},{\dot{q}}_{i})$ is a function of the displacement and velocity generalized coordinates, while the potential energy is a function of the displacement generalized coordinate, i.e., $U(q_{i})$. Thus, the first variation of these two energy terms is $\delta T=\frac{\partial T}{\partial q_{i}}\delta q_{i}+\frac{\partial T}{\partial {\dot{q}}_{i}}\delta {\dot{q}}_{i}$ and $\delta U=\frac{\partial T}{\partial q_{i}}\delta q_{i}$, respectively. Integrating over time and taking into account the end conditions for the generalized coordinates, i.e., $\delta q_{i}\left(t_{0}\right)=\delta q_{i}\left(t_{1}\right)=0$, yields the virtual work statement in the form of $\int_{t_{0}}^{t_{1}}\left\{\frac{\partial T}{\partial q_{i}}\delta q_{i}-\frac{d}{dt}\left(\frac{\partial T}{\partial {\dot{q}}_{i}}\right)\delta q_{i}-\frac{\partial U}{\partial q_{i}}\delta q_{i}+Q_{i}\delta q_{i}-c_{i}{\dot{q}}_{i}\delta q_{i}\right\}dt=0$. Since $\delta q_{i}\neq 0$ in the previous equation is a common factor for the time interval $\left(t_{0},t_{1}\right)$, it cancels out, yielding the Lagrange equations as follows:

(A4) $$\frac{d}{dt}\left(\frac{\partial T}{\partial {\dot{q}}_{i}}\right)-\frac{\partial T}{\partial q_{i}}+\frac{\partial U}{\partial q_{i}}+c_{i}{\dot{q}}_{i}=0$$

## Appendix B

The symbols used are the same as those appearing in the text. Furthermore, the algorithm used is given in [24]; see the following web address: https://mpmath.org/, accessed on 20 October 2023.

**Algorithm A1** Evaluation of the generalized coordiantes of the beam-TMD system	
	Function: TMD
	$Input:\;q_{i}=0,\;{\dot{q}}_{i}=0,\;{\ddot{q}}_{i}=0,t=0,\;t_{b}=L/v,\;t_{f}=1.5,\;dt=1/128$
	Output: Y,Time
1.	$y=[q_{i},{\dot{q}}_{i},\;{\ddot{q}}_{i}]$
2.	Y.append(y)
3.	Time.append(t)
4.	$while\;t<t_{b}+t_{f}:$
5.	t=t+dt
6.	$if\;t\leq t_{b}:$
7.	Moving_Mass=M
8.	else
9.	Moving_Mass=0
10.	$y=iteration\left(M\left(vt\right),C\left(vt\right),K\left(vt\right),F\left(vt\right),y\right)$
11.	Y.append(y)
12.	Time.append(t)
13.	endwhile
	Function: iteration
	$Input:\;M\left(vt\right),C\left(vt\right),K\left(vt\right),F\left(vt\right),y$
	Output: y
1.	$Q_{i}\left(s\right)\;=LaplaceTransform\left(Cramer\left(M\left(vt\right),C\left(vt\right),K\left(vt\right),F\left(vt\right),y\right)\right)$
2.	$q_{i}\left(dt\right)=InvertLaplace\left(Q_{i}\left(s\right),dt,\;method{=\;}^{\prime }talbot^{\prime }\right)$
3.	${\dot{q}}_{i}\left(dt\right)=InvertLaplace\left(sQ_{i}\left(s\right)-q_{i}\left(0\right),dt,\;method{=\;}^{\prime }talbot^{\prime }\right)$
4.	${\ddot{q}}_{i}\left(dt\right)=InvertLaplace\left(s^{2}Q_{i}\left(s\right)-sq_{i}\left(0\right)-{\dot{q}}_{i}\left(0\right),dt,\;method{=\;}^{\prime }talbot^{\prime }\right)$
5.	$y=[q_{i},{\dot{q}}_{i},\;{\ddot{q}}_{i}]$
6.	return y
